# The allelic spectrum of Charcot–Marie–Tooth disease in over 17,000 individuals with neuropathy

**DOI:** 10.1002/mgg3.106

**Published:** 2014-08-21

**Authors:** Christina DiVincenzo, Christopher D Elzinga, Adam C Medeiros, Izabela Karbassi, Jeremiah R Jones, Matthew C Evans, Corey D Braastad, Crystal M Bishop, Malgorzata Jaremko, Zhenyuan Wang, Khalida Liaquat, Carol A Hoffman, Michelle D York, Sat D Batish, James R Lupski, Joseph J Higgins

**Affiliations:** 1 Quest Diagnostics, Athena DiagnosticsMarlborough, Massachusetts; 2 Departments of Molecular and Human Genetics and Pediatrics, Baylor College of MedicineHouston, Texas

**Keywords:** Charcot–Marie–Tooth disease, genetic testing, high-throughput nucleotide sequencing, molecular epidemiology, peripheral neuropathy

## Abstract

We report the frequency, positive rate, and type of mutations in 14 genes (*PMP22*, *GJB1*, *MPZ*, *MFN2*, *SH3TC2*, *GDAP1*, *NEFL*, *LITAF*, *GARS*, *HSPB1*, *FIG4*, *EGR2*, *PRX*, and *RAB7A*) associated with Charcot–Marie–Tooth disease (CMT) in a cohort of 17,880 individuals referred to a commercial genetic testing laboratory. Deidentified results from sequencing assays and multiplex ligation-dependent probe amplification (MLPA) were analyzed including 100,102 Sanger sequencing, 2338 next-generation sequencing (NGS), and 21,990 MLPA assays. Genetic abnormalities were identified in 18.5% (*n* = 3312) of all individuals. Testing by Sanger and MLPA (*n* = 3216) showed that duplications (dup) (56.7%) or deletions (del) (21.9%) in the *PMP22* gene accounted for the majority of positive findings followed by mutations in the *GJB1* (6.7%), *MPZ* (5.3%), and *MFN2* (4.3%) genes. *GJB1* del and mutations in the remaining genes explained 5.3% of the abnormalities. Pathogenic mutations were distributed as follows: missense (70.6%), nonsense (14.3%), frameshift (8.7%), splicing (3.3%), in-frame deletions/insertions (1.8%), initiator methionine mutations (0.8%), and nonstop changes (0.5%). Mutation frequencies, positive rates, and the types of mutations were similar between tests performed by either Sanger (*n* = 17,377) or NGS (*n* = 503). Among patients with a positive genetic finding in a CMT-related gene, 94.9% were positive in one of four genes (*PMP22*, *GJB1*, *MPZ*, or *MFN2*).

## Introduction

Charcot–Marie–Tooth disease (CMT) is a common, clinically heterogeneous group of inherited peripheral neuropathies with an estimated prevalence of 1 in 2500 individuals (Wiszniewski et al. 2013). The salient clinical features include an average onset at age 12, impaired tendon reflexes, a progressive weakness of distal musculature, and abnormalities of the peripheral nerve axon or its adjacent myelin sheath (De Jonghe et al. 1997; Keller and Chance 1999; Nelis et al. 1999). Research studies have shown that CMT is a complex molecular disorder with over a 1000 different putative mutations in 80 disease-associated genes (Timmerman et al. 2014). Approximately 30 genes involved in axonal transport, myelin structure, and membrane metabolism have been found in multiple unrelated families or confirmed by functional studies (Saifi et al. 2003; Saporta et al. 2011). The large spectrum of genetically identifiable disease alleles complicates the molecular diagnosis, but a few genes account for over 90% of known genetic causes (Siskind et al. 2013).

A tiered approach to genetic testing is recommended by the American Academy of Neurology, the American Academy of Neuromuscular and Electrodiagnostic Medicine, the American Academy of Physical Medicine and Rehabilitation (2009 AAN Practice Parameter), based on the disease inheritance pattern, nerve conduction velocities, and the population frequency of specific gene mutations (England et al. 2009a,b). This recommendation relies on a meta-analysis of mutation frequencies reported in previous studies, and includes a decision algorithm with three successive tiers of gene testing prioritized according to attributed risk (England et al. 2009a,b). This tiered process can lead to a laborious and prolonged evaluation when using Sanger sequencing methods. Recent advances in next-generation sequencing (NGS) have streamlined this process by increasing the capability to rapidly and efficiently sequence many genes in a massively parallel manner.

In this study, we analyzed the results of 100,102 Sanger sequencing assays, 2338 NGS, and 21,990 multiplex ligation-dependent amplification (MLPA) dosage analysis assays in 17,880 individuals referred to a commercial laboratory for the diagnostic testing of 14 CMT-related genes. The large size of this cohort provides a rich data source with which to establish robust and accurate mutation frequencies and lends insight into the allelic spectrum of genes involved in neuropathy.

## Methods

### Data mining

The study involved the collection of existing data in such a manner that subjects could not be identified, directly or through identifiers linked to the subjects. The deidentified data were stripped of all protected health information as defined by the Health Insurance Portability and Accountability Act (HIPAA). The results from sequencing assays and dosage analyses were extracted from an internal database by gene name without any identifying information. Ordering healthcare providers provided written attestation that informed consent was obtained before all testing. In all cases, the healthcare providers ordered genetic testing on a standardized requisition form for clinical indications that included inheritance patterns, the presence of a peripheral neuropathy, electrodiagnostic profiles, and variable clinical information. ICD codes were also provided based on pertinent signs and symptoms. Genes were evaluated and selected for their diagnostic capability based on analytic validity, clinical validity, clinical utility, and any potential ethical, legal, and social issues (Haddow and Palomaki 2003; CDC 2007). In contrast to the testing strategy for Sanger which was based on inheritance patterns and the results of nerve conduction studies, NGS was based on the 2009 AAN Practice Parameter tiered approach (England et al. 2009a,b).

### Molecular genetic analysis

#### Sanger sequencing

Sequencing of 14 genes including *EGR2*, *FIG4*, *GARS*, *GDAP1*, *GJB1*, *HSPB1*, *LITAF*, *MFN2*, *MPZ*, *NEFL*, *PMP22*, *PRX*, *RAB7A*, and *SH3TC2* was performed by PCR amplification of exonic sequences and exon–intron boundaries (at least 10 base pairs [bp] into the flanking introns) of purified genomic DNA from peripheral blood followed by automated DNA sequencing on an Applied Biosystems 3730xl DNA Analyzer (ABI, Carlsbad, CA).

#### Next-generation sequencing

NGS was performed on the same 14 genes as Sanger sequencing including *EGR2*, *FIG4*, *GARS*, *GDAP1*, *GJB1*, *HSPB1*, *LITAF*, *MFN2*, *MPZ*, *NEFL*, *PMP22*, *PRX*, *RAB7A*, and *SH3TC2*. Genomic DNA was sheared to an approximate mean fragment length of 200 bp using the Covaris LE220 AFA instrument (Covaris Inc., Woburn, MA). Sheared DNA was used for library preparation of targeted regions using in-solution hybrid capture (Agilent Technologies, Inc., Santa Clara, CA). Approximately 6 *μ*g of sheared genomic DNA from each sample was used for downstream library preparation steps using methods described previously (Rohland and Reich 2012). The bait library was designed using the Agilent eArray with an average bait tiling depth setting of 5×. This library consisted of all coding exons and 20 bases of adjacent intronic regions. Quantitative PCR was used to measure the concentration of viable fragments for sequencing (Quail et al. 2008). The pooled sample library was sequenced on a MiSeq Personal Sequencer (Illumina, San Diego, CA) following the manufacturer's protocol. The sequencing reads were demultiplexed using unique molecular identification sequences (MID) as described previously (Rohland and Reich 2012). Any remaining adapter or MID sequences were removed from the sequencing reads using the fastq-mcf part of ea-utils version 1.1.2 (Aronesty 2011). Sequences from each sample were then aligned to the GRCh37.1 (hg19) genome build using the Burrows–Wheeler Aligner version 0.6.2 (Li and Durbin 2010). Duplicate fragments and sequences flagged as failing filter criteria were excluded from downstream analysis for each sample. Local realignment, base quality score recalibration, and variant calling were performed using the Genome Analysis Toolkit version 2.3.9 (Broad Institute, Cambridge, MA) (DePristo et al. 2011). Variant calling was performed on individual samples rather than on all samples simultaneously. Basic variant annotations were generated using Alamut HT version 1.1.2 (Interactive Biosoftware, Rouen, France) and were further refined and screened for accuracy utilizing a custom database created for NGS data review.

#### Multiplex ligation-dependent probe amplification

MLPA (Armour et al. 2000; den Dunnen and White 2006) was used to test for *PMP22* dup/del and *GJB1* del on genomic DNA extracted from whole blood. Single exon deletions were analyzed by Sanger sequencing when possible to rule out a false-positive test result due to a sequence variant interfering with the probe annealing site.

#### Pathogenicity assessment

A standardized pathogenicity assessment scoring process was used to categorize variants according to their assessed likelihood of pathogenicity, based on evaluation of multiple independent types of evidence. Categories included known pathogenic variants, known normal variants, and subcategories for variants of unknown significance having varying likelihoods of pathogenicity. This scoring system conforms to ACMG guidelines recommending multiple independent lines of evidence in order to classify a variant as benign or pathogenic (Richards et al. 2008).

### Mutation frequency and positive rate calculations

The frequencies of pathogenic mutations and nonsynonymous variants of unknown significance (VUS) in 14 CMT genes were determined by reviewing the results of genetic tests performed during a period between 2009 and 2013 at a CLIA/CAP certified commercial laboratory. Mutations were interpreted as pathogenic if supported by published functional or segregation studies. Nonsense, frameshift, and canonical splice site mutations were considered likely loss of function pathogenic alleles. Mutations to the start and stop codons were also interpreted as pathogenic. Patients with a single recessive mutation and a VUS were considered carriers and were not included in mutation frequency and positive rate calculations.

In order to compare the results with the 2009 AAN Practice Parameter (England et al. 2009a,b) and a similar study (Murphy et al. 2012), the mutation frequency and positive rates were calculated for the time period between 2009 and 2013 using Sanger sequencing and MLPA. The positive rates were calculated based on the total number of patients tested for each CMT gene. This calculation considered that all 14 genes were not tested in every patient. The mutation frequency was determined by calculating the percentage of positive results attributed to each gene out of the total number of genetically positive patients. Analyses using a combination of NGS and MLPA were conducted separately. A two-tailed Fisher's exact test was used to analyze contingency tables comparing the differences in the positive rate between Sanger and NGS methods.

## Results

### Mutation frequencies

**Figure 1:**
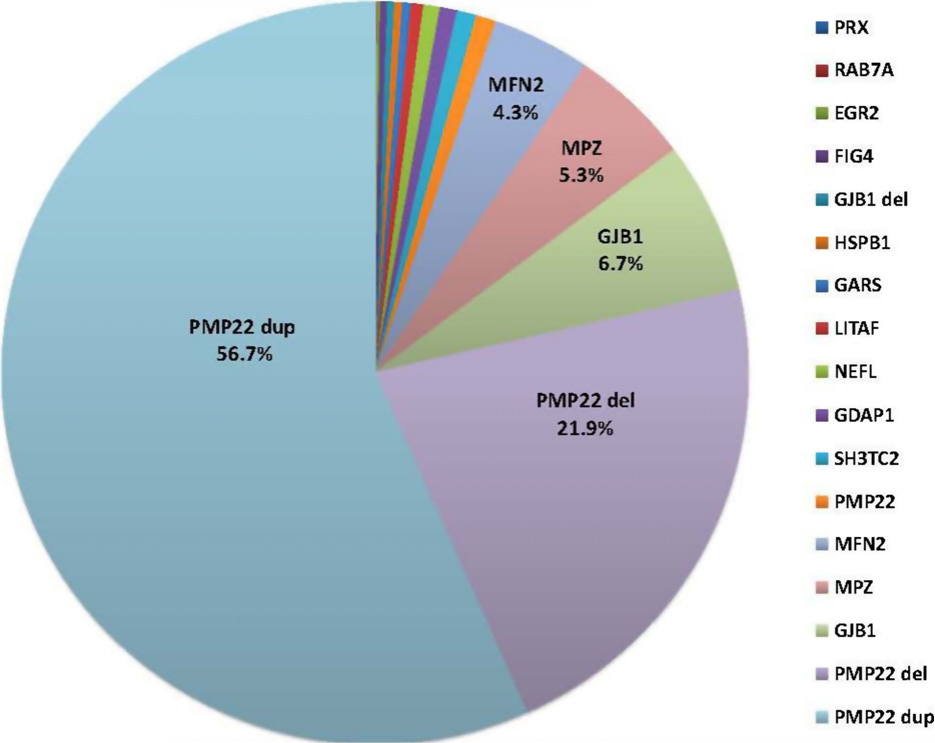
The mutation frequency of Charcot–Marie–Tooth disease genes in a large cohort (*n* = 17,377) analyzed at a commercial laboratory. The pie chart shows the percentage of positive results attributed to each gene by color out of the total number of genetically positive patients (*n* = 3216) as determined by Sanger sequencing and MLPA. Mutations in four genes (*PMP22* dup/del, *GJB1*, *MPZ*, and *MFN2*) accounted for 94.9% of the genetically positive patients in our cohort. PMP22 duplications (dup) accounted 56.7% of positive patients, *PMP22* deletions (del) 21.9%, *GJB1* 6.7%, *MPZ* 5.3%, *MFN2* 4.3%, *PMP22* 0.9%, *SH3TC2* 0.8%, *GDAP1* 0.7%, *NEFL* 0.7%, *LITAF* 0.5%, *GARS* 0.4%, *HSPB1* 0.3%, *GJB1* del 0.3%, *FIG4* 0.3%, *EGR2* 0.1%, *RAB7A* 0.1%, and *PRX* 0.03%.

The data included 100,102 Sanger sequencing assays, 2338 NGS, and 21,990 MLPA assays in 17,880 individuals referred to a commercial laboratory for diagnostic testing of 14 CMT-related genes. FigureFigure1 shows the allele frequency distribution of mutation positive patients (*n* = 3216) identified using both Sanger sequencing and dosage analysis techniques performed on 14 genes in 17,377 patient samples sent to a commercial laboratory between 2009 and 2013. *PMP22* duplication and deletion copy number variations (CNVs) accounted for the majority of pathogenic mutations (56.7% and 21.9%, respectively) followed by nucleotide variation mutations (SNV) in *GJB1* (6.7%), *MPZ* (5.3%), and *MFN2* (4.3%). These four genes accounted for 94.9% of genetically positive patients in this cohort. NGS and dosage analysis techniques performed on the same 14 genes in 503 patients showed similar mutation frequencies (Table S1). Table1 compares the mutation frequencies between the current study and the 2009 AAN Practice Parameter (England et al. 2009a,b). Although mutation frequencies were similar for *MPZ*, differences were found between *PMP22* CNVs (78.6% vs. 70%) and *GJB*1 (6.7% vs. 12.0%), and *MFN2* (4.3% of CMT vs. 33.0% CMT2;9.3% CMT). *PMP22* del were not described in the 2009 AAN Practice Parameter, but del or HNPP deletion is not uncommonly found presenting with a classic CMT phenotype particularly in older individuals. Mutations were less frequent in other genes associated with CMT including SNV of *PMP22* (0.9%), *SH3TC2* (0.8%), *GDAP1* (0.7%), *NEFL* (0.7%), *LITAF* (0.5%), *GARS* (0.4%), *HSPB1* (0.3%), *GJB1* deletion CNV (0.3%), *FIG4* (0.3%), *EGR2* (0.1%), *RAB7A* (0.1%), and *PRX* (0.03%). (Fig.1). Thus, recessive CMT loci *SH3TC2*, *GDAP1*, *PRX*, and *FIG4* contributed to a small fraction of neuropathy subjects.

**Table 1:** A comparison of the current study and the 2009 AAN Practice Parameter by England et al. of Charcot–Marie–Tooth mutation frequencies in four common genes

Gene	Current study (% positive)	2009 AAN Practice Parameter (% positive)
*PMP22* CNVs	78.6	70.0
*GJB1*	6.7	12.0
*MPZ*	5.3	5.0
*MFN2*	4.3	9.3

CNVs, copy number variations including duplication and deletion; *PMP22*, peripheral myelin protein 22; *GJB1*, gap junction protein, *β*1; *MPZ*, myelin protein zero; *MFN2*, mitofusin 2; *SH3TC2*, SH3 domain and tetratricopeptide repeat domain 2; *GDAP1*, ganglioside-induced differentiation-associated protein; *NEFL*, neurofilament protein light polypeptide; *LITAF*, lipopolysaccharide-induced tumor necrosis factor *α* factor; *GARS*, glycyl-tRNA synthetase; *HSPB1*, heat-shock 27-kd protein 1; *FIG4*, *Saccharomyces cerevisiae* homolog of fig4; *EGR2*, early growth response 2; *PRX*, periaxin; *RAB7A*, RAS-associated protein RAB7.

### Positive rates

The positive rates for mutations in 14 CMT genes including VUS are available in Table S2, S3 (Sanger), and S4 (NGS). Dosage analyses of *PMP22* indicated a positive rate of 10.49% for dup and 4.06% for del. Sanger sequencing yielded low positive rates for *GJB1* (1.23%), *MFN2* (0.79%), *MPZ* (0.97%), and other genes (≤0.16%). Nonsynonymous VUS were most frequent in *PRX* (2.62%), *SH3TC2* (1.97%), and *MFN2* (1.12%), and less often in the other genes (≤0.77%) (Table S3). Comparative analysis between the positive rate of CMT mutations identified in the current study (*n* = 17,377) and another study performed in a diagnostic setting (Murphy et al. 2012) (*n* = 1182) (Table2) reveal that the most striking differences were in the higher positive rates of *PMP22* CNVs, *GJB1*, and *MFN2*. Differences in positive rates were 1.4-fold for *PMP22* dup/del (20.90% vs. 14.54%), sevenfold for *GJB1* (8.50% vs. 1.23%), and fivefold for *MFN2* (4.10% vs. 0.79%).

**Table 2:** A comparison of the positive rates detected in Charcot–Marie–Tooth diagnostic cases between the current study (*n* = 17,377) and Murphy et al. (*n* = 1182)

Gene	Current study (% positive)	Murphy et al. (% positive)^1^
*PMP22* CNVs	14.54	20.9
*GJB1*	1.23	8.50
*MPZ*	0.97	1.50
*MFN2*	0.79	4.10
*SH3TC2*	0.16	0.30
*GDAP1*	0.14	0.80
*NEFL*	0.13	0.20
*PMP22*	0.16	0.40
*LITAF*	0.10	0.20
*GARS*	0.07	NA
*FIG4*	0.06	NA
*HSPB1*	0.06	0.10
*GJB1 del*	0.06	NA
*EGR2*	0.02	0.40
*PRX*	0.01	NA
*RAB7A*	0.01	NA

CNVs, copy number variations including duplication and deletion; NA, not available; *PMP22*, peripheral myelin protein 22; *GJB1*, gap junction protein, *β*1; *MPZ*, myelin protein zero; *MFN2*, mitofusin 2; *SH3TC2*, SH3 domain and tetratricopeptide repeat domain 2; *GDAP1*, ganglioside-induced differentiation-associated protein; *NEFL*, neurofilament protein light polypeptide; *LITAF*, lipopolysaccharide-induced tumor necrosis factor *α* factor; *GARS*, glycyl-tRNA synthetase; *HSPB1*, heat-shock 27-kd protein 1; *FIG4*, *Saccharomyces cerevisiae* homolog of fig4; *EGR2*, early growth response 2; *PRX*, periaxin; *RAB7A*, RAS-associated protein RAB7.

1 Genetic diagnoses in patients with Charcot–Marie–Tooth disease not attending an inherited neuropathy clinic.

### Types of pathogenic mutations

**Figure 2:**
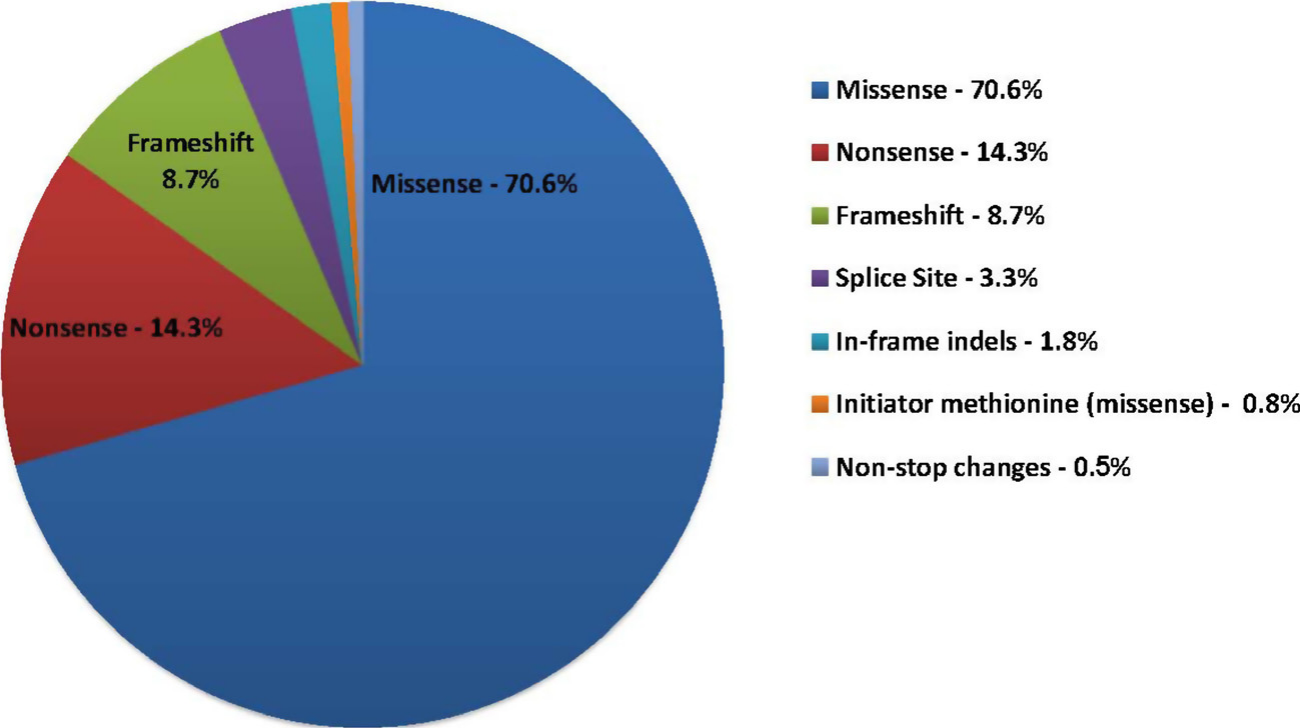
The distribution of pathogenic sequencing mutation types in Charcot–Marie–Tooth disease. The pie chart shows the percentage of mutation types by color out of the total number of genetically positive patients (*n* = 3216) as determined by Sanger sequencing and MLPA. Missense mutations accounted for the majority (70.6%) of pathogenic sequencing variants identified in this cohort, followed by nonsense 14.3%, frameshift 8.7%, and splice site 3.3% mutations. Other mutation types accounted for the remaining 3.1% of pathogenic mutations.

**Figure 3:**
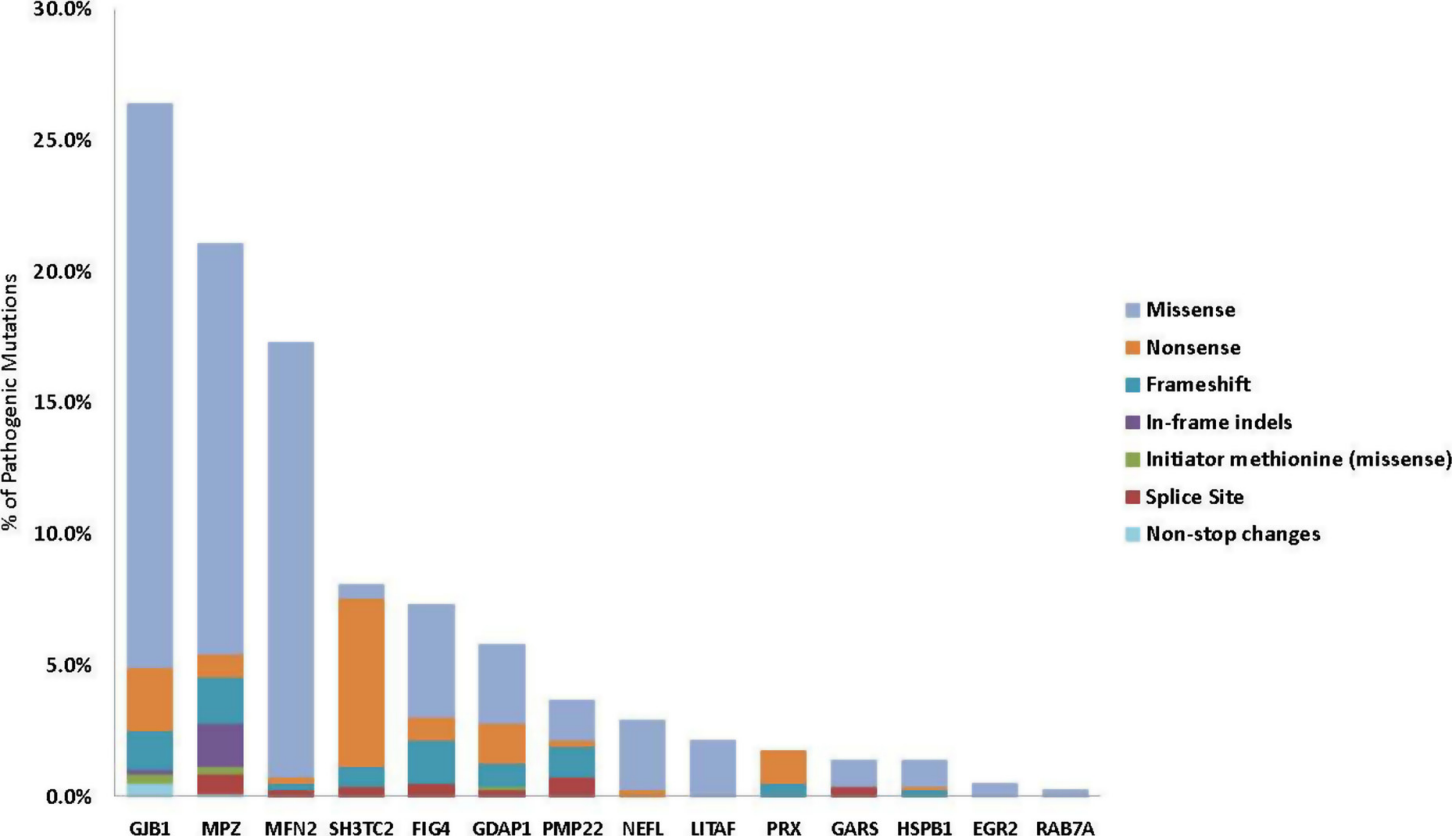
The distribution of pathogenic sequencing mutation types per Charcot–Marie–Tooth disease gene. The bar graph shows the distribution of mutation types by color for each CMT gene. Missense mutations accounted for a majority of pathogenic variants identified in most genes. Pathogenic mutations identified in the *SH3TC2* and *PRX* genes were mostly nonsense mutations.

Missense (70.6%), nonsense (14.3%), frameshift (8.7%), and splice site (3.3%) mutations accounted for 96.8% of pathogenic sequencing mutations (including carrier status patients). Other mutations including in-frame indels (1.8%), initiator methionine (0.8%), and nonstop changes (0.5%) were observed less frequently (Fig.2). Eighty-seven previously unpublished predicted pathogenic mutations were identified in the *MPZ* (18), *SH3TC2* (14), *GJB1* (12), *GDAP1* (9), *FIG4* (7), *PMP22* (7), *PRX* (7), *MFN2* (6), *GARS* (3), *HSPB1* (2), and *NEFL* (2) genes clearly documenting the contributions of rare variants to neuropathy. Of all new predicted pathogenic variants identified, frameshift mutations accounted for 42.5%, nonsense mutations for 28.7%, and splice site mutations for 20.7%. The remaining newly identified mutations were split among changes to the initiator methionine (3.4%), nonstop changes (3.4%), and missense changes (1.1%) (Table S5). Five mutations accounted for 20.3% of all the pathogenic mutations in this study, with two mutations in *MFN2* (c.2219G>C, p.W740S; c.227T>C; p.L76P) comprising 8.7%. Other frequent mutations included *SH3TC2* (c.2860C>T; p.Arg954* [4.8%]), *FIG4* (c.122T>C; p.Ile41Thr [4.3%]), and *GJB1* (c.305A>G; p.Glu102Gly [2.5%]) (Table S6). The distribution of pathogenic sequencing mutation types in each CMT gene is illustrated in Figure3. Missense mutations accounted for the majority of pathogenic variants in most genes. Most pathogenic mutations identified in the *SH3TC2* and *PRX* genes were nonsense alleles consistent with a loss of function at these loci.

### Variants of unknown significance

VUS were detected in all genes included in this study (Table S2). Nonsynonymous VUS were identified most frequently by Sanger sequencing in *PRX* (2.62%), *SH3TC2* (1.97%), and *MFN2* (1.12%) (Table S3). Similar rates for nonsynonymous VUS were identified by NGS (Table S4).

### Comparison of the positive rates between Sanger and NGS

We compared the frequency of positive results of 121,561 molecular tests performed by Sanger sequencing (100,102) and MLPA (21,459) between 2009 and 2013 in 17,377 individuals (Table S3) to 2869 molecular tests performed in 503 individuals by NGS sequencing (2388) and MLPA (531) in 2013 (Table S4). The frequency of positive results for 14 CMT genes was not significantly different (*P* < 0.05) (Table S7) despite differences in the testing strategy between Sanger sequencing and NGS.

## Discussion

We conducted a study describing data derived from 124,430 individual CMT genetic tests performed on 17,880 patients referred to a clinical diagnostic laboratory. The results provide valuable insight into the current state of genetic diagnostic testing for CMT. The data from our study can be used to draft a coherent policy for population-based carrier testing in individuals with CMT in the general U.S. population. Currently, the 2009 AAN Practice Parameter recommends a tiered reflexive approach to genetic screening for CMT. Currently, no recommendation exists when the family history is unclear or unknown (Boerkoel et al. 2002; Marques et al. 2005). Guidance is needed because 20% of CMT cases may be sporadic (Marques et al. 2005). While up to 90% of sporadic well-defined CMT1 can be due to de novo *PMP22* duplications (Boerkoel et al. 2002; Marques et al. 2005), our data show that mutations in *GJB1*, *MFN2*, *MPZ*, and *PMP22* dup/del comprised 94.9% of positive genetic results supporting a potential recommendation to perform initial genetic testing of these genes based solely on clinical phenotype. While these four genes cause the majority of CMT with a known genetic etiology, other loci also cause CMT, challenging physicians to obtain an unambiguous genetic diagnosis (Siskind et al. 2013). In approximately 1 in 20 cases, a more detailed clinical evaluation including electrodiagnostic studies (England et al. 2009a,b) may clarify the selection of other more rarely found mutated CMT genes.

The differences in mutation frequencies and positive rates between our study and others (*n* = 3319) (Saporta et al. 2011; Murphy et al. 2012; Sivera et al. 2013) highlight discrepancies in the diagnostic approaches to the genetic evaluation of CMT, as well as ascertainment differences. For example, the positive rate in other studies for *PMP22* dup/del ranged from 48.8% to 63.2% followed by mutations in *GJB1* (range = 14.9–17.3), *MPZ* (range = 4.9–8.5%), and *MFN2* (range = 1.6–4.5%). The frequency of *GDAP1* (11.1%) mutations was higher in a Spanish population (Sivera et al. 2013) compared to our study (0.7%) and other studies (range = 0.8–1.2%) (Saporta et al. 2011; Murphy et al. 2012). Despite the methodological differences between these studies, the rank order of mutation frequencies from the highest to the lowest was similar to our study (i.e., *PMP22* dup/del, *GJB1*, *MPZ*, and *MFN2*). One possible explanation for the lower rate of detecting a genetic cause for CMT in this study as compared to others is the differing levels of referral bias. The population referred to our laboratory for testing may contain a greater number of patients with a borderline, subclinical, or otherwise noncanonical CMT presentation as compared to other studies that describe cohorts with clear CMT phenotypes. Our population is similar to the external population described by Murphy et al. (2012) since the study took place in a diagnostic setting with limited clinical information. In our study, the information (e.g., ICD codes, family history, electrodiagnostic results, presence of a peripheral neuropathy, and hereditary neuropathy test code) provided by ordering healthcare providers indicated a phenotype consistent with CMT. The relatively low number of novel missense changes considered pathogenic in our dataset is most likely due to the stringent requirements for supporting evidence in scoring missense variants as pathogenic relative to other mutation types. This type of VUS interpretation is usually more conservative in the commercial setting in contrast to the research environment. For example, *MFN2* yielded one of the highest discrepancies in positive rates between our study and the study conducted by Murphy et al. (Table2). Pathogenic variants in *MFN2* are typically inherited in an autosomal dominant inheritance pattern, and are usually missense variants. We find that *MFN2* has a high rate of VUS detection, indicating that the interpretation of these variants possibly contributed to the discrepancy found between the studies.

The results of our study suggest that our mutation frequencies and positive rates will not change as we transition from Sanger to NGS technology. Although NGS technology facilitates the simultaneous evaluation of multiple genes, the potential for more VUS when more genes are analyzed may confound interpretation. Whole exome (Lupski et al. 2013) and whole genome studies (Gonzaga-Jauregui et al. 2012) may be useful in identifying new genetic etiologies for CMT phenotypes, but our study supports that targeted sequencing is likely to yield interpretable genetic results in at least 18.5% of CMT patients. The results of our study in a population in over 17,000 individuals support the initial genetic testing of four genes (*PMP22*, *GJB1*, *MPZ*, and *MFN2*) followed by an evaluation of rarer genetic causes in the diagnostic evaluation of CMT.

## Supporting Information

**Table S1.** A comparison of the mutation frequencies attributed to each Charcot–Marie–Tooth gene before and after the introduction of NGS. **Table S2.** Charcot–Marie–Tooth genetic variants scored as variants of unknown significance. **Table S3.** The positive rate for Charcot–Marie–Tooth disease gene mutations and nonsynonymous variants as determined by MLPA and Sanger DNA sequencing (*n* = 17,377). **Table S4.** The positive rate for Charcot–Marie–Tooth disease gene mutations and nonsynonymous variants as determined by MLPA and NGS (*n* = 503). **Table S5.** Previously unpublished pathogenic Charcot–Marie–Tooth mutations detected in this study (*n* = 87). **Table S6.** The top five (20.3%) recurring Charcot–Marie–Tooth mutations in this study. **Table S7.** A comparison of positive rates before and after the introduction of NGS.
