# Supplementary material for: The allelic spectrum of Charcot–Marie–Tooth disease in over 17,000 individuals with neuropathy
Source: Mol Genet Genomic Med. 2014 Aug 21;2(6):522–9. doi: 10.1002/mgg3.106 (PMC4303222; doi:10.1002/mgg3.106)
Supplement: Supplementary file 1 — Table S1. A comparison of the mutation frequencies attributed to each Charcot–Marie–Tooth gene before and after the introduction of NGS. Table S2. Charcot–Marie–Tooth genetic variants scored as variants of unknown significance. Table S3. The positive rate for Charcot–Marie–Tooth disease gene mutations and nonsynonymous variants as determined by MLPA and Sanger DNA sequencing (n = 17,377). Table S4. The positive rate for Charcot–Marie–Tooth disease gene mutations and nonsynonymous variants as determined by MLPA and NGS (n = 503). Table S5. Previously unpublished pathogenic Charcot–Marie–Tooth mutations detected in this study (n = 87). Table S6. The top five (20.3%) recurring Charcot–Marie–Tooth mutations in this study. Table S7. A comparison of positive rates before and after the introduction of NGS. [file mgg30002-0522-sd1.docx]

**Supplemental Materials**

**The Allelic Spectrum of Charcot-Marie-Tooth Disease in Over 17,000 Individuals with Neuropathy**

Christina DiVincenzo,^1†^ Christopher D. Elzinga,^1†^ Adam C. Medeiros, ^1†^ Izabela Karbassi,^1^ Jeremiah Jones,^1^ Matthew Evans,^1^ Corey D. Braastad,^1^ Crystal Bishop,^1^ Malgorzata Jaremko,^1^ Zhenyuan Wang,^1^ Khalida Liaquat,^1^ Carol Hoffman,^1^ Michelle York,^1^ Sat D. Batish,^1^ James R. Lupski,^2^ and Joseph J. Higgins,^1*^

^1^Quest Diagnostics, Athena Diagnostics, Marlborough, Massachusetts; ^2^Department of Molecular and Human Genetics, Department of Pediatrics, Baylor College of Medicine, Houston, Texas.

^†^These authors contributed equally to this work.

*Correspondence to: Joseph J. Higgins, Quest Diagnostics, Athena Diagnostics, 200 Forest Street, Marlborough, MA 01752. E-mail: [Joseph.J.Higgins@QuestDiagnostics.com](mailto:Joseph.J.Higgins@QuestDiagnostics.com)

Contract grant sponsor: Quest Diagnostics

**Supplemental Table 1.** A Comparison of the Mutation Frequencies Attributed to Each Charcot-Marie-Tooth Gene Before and After the Introduction of NGS.

| Gene | Sanger Sequencing*  % of total positives | NGS Sequencing†  % of total positives |
| --- | --- | --- |
| *PMP22 dup* | 56.7 | 65.2 |
| *PMP22 del* | 21.9 | 16.8 |
| *GJB1* | 6.7 | 5.3 |
| *MPZ* | 5.3 | 2.1 |
| *MFN2* | 4.3 | 6.3 |
| *SH3TC2* | 0.9 | 2.1 |
| *PMP22* | 0.8 | 0.0 |
| *GDAP1* | 0.7 | 1.1 |
| *NEFL* | 0.7 | 0.0 |
| *LITAF* | 0.5 | 1.1 |
| *GARS* | 0.4 | 0.0 |
| *HSPB1* | 0.3 | 0.0 |
| *FIG4* | 0.3 | 0.0 |
| *GJB1 del* | 0.3 | 0.0 |
| *EGR2* | 0.1 | 0.0 |
| *PRX* | 0.0 | 0.0 |
| *RAB7A* | 0.1 | 0.0 |

*3,216 positive of 17,377 individuals

†95 positive of 503 individuals

Abbreviations: NGS = next generation DNA sequencing; dup = duplication; del = deletion; *PMP22 =* peripheral myelin protein 22; *GJB1 =* gap junction protein, beta-1; *MPZ =* myelin protein zero; *MFN2 =* mitofusin 2*; SH3TC2 =* SH3 domain and tetratricopeptide repeat domain 2; *GDAP1 =* ganglioside-induced differentiation-associated protein; *NEFL =* neurofilament protein light polypeptide; *LITAF =* lipopolysaccharide-induced tumor necrosis factor-alpha factor; *GARS =* glycyl-tRNA synthetase, *HSPB1* heat-shock 27-kd protein 1; *FIG4 =* S. Cerevisiae homolog of fig4; *EGR2* = early growth response 2; *PRX =* periaxin*; RAB7A =* RAS-associated protein RAB7.

**Supplemental Table 2.** Charcot-Marie-Tooth Genetic Variants Scored as Variants of Unknown Significance.

| Gene | Reference Sequence | Variant | Mutation Type |
| --- | --- | --- | --- |
| *EGR2* | NM_000399.3 | c.52G>T; p.Val18Leu | Missense |
| *EGR2* | NM_000399.3 | c.115T>C; p.Phe39Leu | Missense |
| *EGR2* | NM_000399.3 | c.192G>C; p.Met64Ile | Missense |
| *EGR2* | NM_000399.3 | c.480C>T | Synonymous |
| *EGR2* | NM_000399.3 | c.665T>C; p.Met222Thr | Missense |
| *EGR2* | NM_000399.3 | c.867C>T | Synonymous |
| *EGR2* | NM_000399.3 | c.877C>T; p.Arg293Trp | Missense |
| *EGR2* | NM_000399.3 | c.906_911dup; p.Ala308_Ala309dup | mRNA reading frame maintained |
| *EGR2* | NM_000399.3 | c.915C>A | Synonymous |
| *EGR2* | NM_000399.3 | c.925G>A; p.Ala309Thr | Missense |
| *EGR2* | NM_000399.3 | c.1090C>T; p.His364Tyr | Missense |
| *EGR2* | NM_000399.3 | c.1179C>T | Synonymous |
| *EGR2* | NM_000399.3 | c.1226G>A; p.Arg409Gln | Missense |
| *EGR2* | NM_000399.3 | c.1352G>T; p.Gly451Val | Missense |
| *EGR2* | NM_000399.3 | c.1399G>C; p.Ala467Pro | Missense |
| *FIG4* | NM_014845.5 | c.14C>T; p.Ala5Val | Missense |
| *FIG4* | NM_014845.5 | c.42G>A | Synonymous |
| *FIG4* | NM_014845.5 | c.52T>C; p.Tyr18His | Missense |
| *FIG4* | NM_014845.5 | c.53A>G; p.Tyr18Cys | Missense |
| *FIG4* | NM_014845.5 | c.101C>T; p.Thr34Met | Missense |
| *FIG4* | NM_014845.5 | c.173A>G; p.Tyr58Cys | Missense |
| *FIG4* | NM_014845.5 | c.289+4A>G | Intronic |
| *FIG4* | NM_014845.5 | c.309A>C; p.Glu103Asp | Missense |
| *FIG4* | NM_014845.5 | c.594A>C; p.Gln198His | Missense |
| *FIG4* | NM_014845.5 | c.730C>T; p.Arg244Cys | Missense |
| *FIG4* | NM_014845.5 | c.815T>C; p.Ile272Thr | Missense |
| *FIG4* | NM_014845.5 | c.834A>T; p.Lys278Asn | Missense |
| *FIG4* | NM_014845.5 | c.1091T>A; p.Met364Lys | Missense |
| *FIG4* | NM_014845.5 | c.1137+5G>T | Intronic |
| *FIG4* | NM_014845.5 | c.1187C>G; p.Ala396Gly | Missense |
| *FIG4* | NM_014845.5 | c.1271+5A>G | Intronic |
| *FIG4* | NM_014845.5 | c.1272-10C>G | Intronic |
| *FIG4* | NM_014845.5 | c.1305G>C | Synonymous |
| *FIG4* | NM_014845.5 | c.1405G>A; p.Gly469Arg | Missense |
| *FIG4* | NM_014845.5 | c.1574A>G; p.Asp525Gly | Missense |
| *FIG4* | NM_014845.5 | c.1583+3A>G | Intronic |
| *FIG4* | NM_014845.5 | c.1584-8T>A | Intronic |
| *FIG4* | NM_014845.5 | c.1940A>G; p.Tyr647Cys | Missense |
| *FIG4* | NM_014845.5 | c.1949-10T>G | Intronic |
| *FIG4* | NM_014845.5 | c.2095C>T; p.Arg699Cys | Missense |
| *FIG4* | NM_014845.5 | c.2096G>A; p.Arg699His | Missense |
| *FIG4* | NM_014845.5 | c.2097-10C>T | Intronic |
| *FIG4* | NM_014845.5 | c.2147G>A; p.Arg716His | Missense |
| *FIG4* | NM_014845.5 | c.2241G>A | Synonymous |
| *FIG4* | NM_014845.5 | c.2376+5G>C | Intronic |
| *FIG4* | NM_014845.5 | c.2377-10T>A | Intronic |
| *FIG4* | NM_014845.5 | c.2444T>C; p.Phe815Ser | Missense |
| *FIG4* | NM_014845.5 | c.2547-5T>G | Intronic |
| *FIG4* | NM_014845.5 | c.2599A>G; p.Arg867Gly | Missense |
| *FIG4* | NM_014845.5 | c.2660T>C; p.Met887Thr | Missense |
| *FIG4* | NM_014845.5 | c.2690T>C; p.Met897Thr | Missense |
| *GARS* | NM_002047.2 | c.51G>A | Synonymous |
| *GARS* | NM_002047.2 | c.56_61dup; p.Leu19_Leu20dup | mRNA reading frame maintained |
| *GARS* | NM_002047.2 | c.236G>A; p.Arg79Gln | Missense |
| *GARS* | NM_002047.2 | c.253A>G; p.Lys85Glu | Missense |
| *GARS* | NM_002047.2 | c.270C>T | Synonymous |
| *GARS* | NM_002047.2 | c.302G>A; p.Arg101His | Missense |
| *GARS* | NM_002047.2 | c.332C>T; p.Ala111Val | Missense |
| *GARS* | NM_002047.2 | c.349G>A; p.Asp117Asn | Missense |
| *GARS* | NM_002047.2 | c.401A>G; p.Tyr134Cys | Missense |
| *GARS* | NM_002047.2 | c.408A>G | Synonymous |
| *GARS* | NM_002047.2 | c.446A>G; p.Asp149Gly | Missense |
| *GARS* | NM_002047.2 | c.534C>T | Synonymous |
| *GARS* | NM_002047.2 | c.631T>G; p.Cys211Gly | Missense |
| *GARS* | NM_002047.2 | c.647A>G; p.His216Arg | Missense |
| *GARS* | NM_002047.2 | c.699C>T | Synonymous |
| *GARS* | NM_002047.2 | c.787G>A; p.Val263Ile | Missense |
| *GARS* | NM_002047.2 | c.816A>G | Synonymous |
| *GARS* | NM_002047.2 | c.855C>G; p.Phe285Leu | Missense |
| *GARS* | NM_002047.2 | c.1100A>G; p.Asn367Ser | Missense |
| *GARS* | NM_002047.2 | c.1140C>G | Synonymous |
| *GARS* | NM_002047.2 | c.1162C>T; p.Arg388Trp | Missense |
| *GARS* | NM_002047.2 | c.1358A>G; p.Tyr453Cys | Missense |
| *GARS* | NM_002047.2 | c.1420C>A | Synonymous |
| *GARS* | NM_002047.2 | c.1600C>T | Synonymous |
| *GARS* | NM_002047.2 | c.1613+9T>A | Intronic |
| *GARS* | NM_002047.2 | c.1614-4G>C | Intronic |
| *GARS* | NM_002047.2 | c.1660G>A; p.Asp554Asn | Missense |
| *GARS* | NM_002047.2 | c.1716G>A | Synonymous |
| *GARS* | NM_002047.2 | c.1743G>C | Synonymous |
| *GARS* | NM_002047.2 | c.1809A>G | Synonymous |
| *GARS* | NM_002047.2 | c.1828G>A; p.Val610Ile | Missense |
| *GARS* | NM_002047.2 | c.1852G>A; p.Val618Ile | Missense |
| *GARS* | NM_002047.2 | c.1923T>C | Synonymous |
| *GARS* | NM_002047.2 | c.1929A>G | Synonymous |
| *GARS* | NM_002047.2 | c.1991T>C; p.Ile664Thr | Missense |
| *GARS* | NM_002047.2 | c.2039A>G; p.Tyr680Cys | Missense |
| *GARS* | NM_002047.2 | c.2065C>G; p.Arg689Gly | Missense |
| *GDAP1* | NM_018972.2 | c.18A>G | Synonymous |
| *GDAP1* | NM_018972.2 | c.90T>C | Synonymous |
| *GDAP1* | NM_018972.2 | c.102C>G | Synonymous |
| *GDAP1* | NM_018972.2 | c.116A>G; p.Lys39Arg | Missense |
| *GDAP1* | NM_018972.2 | c.117+10G>A | Intronic |
| *GDAP1* | NM_018972.2 | c.248G>C; p.Gly83Ala | Missense |
| *GDAP1* | NM_018972.2 | c.283G>C; p.Asp95His | Missense |
| *GDAP1* | NM_018972.2 | c.287A>G; p.Tyr96Cys | Missense |
| *GDAP1* | NM_018972.2 | c.345C>G; p.Ser115Arg | Missense |
| *GDAP1* | NM_018972.2 | c.346A>T; p.Met116Leu | Missense |
| *GDAP1* | NM_018972.2 | c.399G>A; p.Met133Ile | Missense |
| *GDAP1* | NM_018972.2 | c.419G>T; p.Cys140Phe | Missense |
| *GDAP1* | NM_018972.2 | c.458C>T; p.Pro153Leu | Missense |
| *GDAP1* | NM_018972.2 | c.693A>T | Synonymous |
| *GDAP1* | NM_018972.2 | c.695-9T>A | Intronic |
| *GDAP1* | NM_018972.2 | c.719G>C; p.Cys240Ser | Missense |
| *GDAP1* | NM_018972.2 | c.720C>T | Synonymous |
| *GDAP1* | NM_018972.2 | c.776A>G; p.Lys259Arg | Missense |
| *GDAP1* | NM_018972.2 | c.782T>C; p.Leu261Pro | Missense |
| *GDAP1* | NM_018972.2 | c.839A>C; p.Tyr280Ser | Missense |
| *GDAP1* | NM_018972.2 | c.845G>A; p.Arg282His | Missense |
| *GDAP1* | NM_018972.2 | c.846T>A | Synonymous |
| *GDAP1* | NM_018972.2 | c.894T>C | Synonymous |
| *GDAP1* | NM_018972.2 | c.919A>G; p.Thr307Ala | Missense |
| *GDAP1* | NM_018972.2 | c.965C>T; p.Thr322Met | Missense |
| *GDAP1* | NM_018972.2 | c.1006G>T; p.Ala336Ser | Missense |
| *GDAP1* | NM_018972.2 | c.1039A>G; p.Met347Val | Missense |
| *GDAP1* | NM_018972.2 | c.1045T>C | Synonymous |
| *GDAP1* | NM_018972.2 | c.1066A>G; p.Asn356Asp | Missense |
| *GJB1* | NM_000166.5 | c.-6G>A | 5'UTR |
| *GJB1* | NM_000166.5 | c.108G>T | Synonymous |
| *GJB1* | NM_000166.5 | c.113T>G; p.Val38Gly | Missense |
| *GJB1* | NM_000166.5 | c.130T>C; p.Trp44Arg | Missense |
| *GJB1* | NM_000166.5 | c.136G>A; p.Asp46Asn | Missense |
| *GJB1* | NM_000166.5 | c.151T>C; p.Phe51Leu | Missense |
| *GJB1* | NM_000166.5 | c.179G>A; p.Cys60Tyr | Missense |
| *GJB1* | NM_000166.5 | c.185G>C; p.Ser62Thr | Missense |
| *GJB1* | NM_000166.5 | c.188T>A; p.Val63Asp | Missense |
| *GJB1* | NM_000166.5 | c.297_299dup; p.Gln99dup | mRNA reading frame maintained |
| *GJB1* | NM_000166.5 | c.302T>C; p.Ile101Thr | Missense |
| *GJB1* | NM_000166.5 | c.318A>G | Synonymous |
| *GJB1* | NM_000166.5 | c.376C>T; p.His126Tyr | Missense |
| *GJB1* | NM_000166.5 | c.412A>G; p.Ser138Gly | Missense |
| *GJB1* | NM_000166.5 | c.462T>C | Synonymous |
| *GJB1* | NM_000166.5 | c.465G>T | Synonymous |
| *GJB1* | NM_000166.5 | c.527C>T; p.Thr176Ile | Missense |
| *GJB1* | NM_000166.5 | c.548G>T; p.Arg183Leu | Missense |
| *GJB1* | NM_000166.5 | c.592T>C; p.Ser198Pro | Missense |
| *GJB1* | NM_000166.5 | c.606C>T | Synonymous |
| *GJB1* | NM_000166.5 | c.617T>G; p.Val206Gly | Missense |
| *GJB1* | NM_000166.5 | c.637A>G; p.Ile213Val | Missense |
| *GJB1* | NM_000166.5 | c.671G>A; p.Arg224His | Missense |
| *GJB1* | NM_000166.5 | c.688C>T; p.Arg230Cys | Missense |
| *GJB1* | NM_000166.5 | c.727T>C; p.Tyr243His | Missense |
| *GJB1* | NM_000166.5 | c.811G>A; p.Ala271Thr | Missense |
| *GJB1* | NM_000166.5 | c.841T>C; p.Ser281Pro | Missense |
| *GJB1* | NM_000166.5 | c.979G>A; p.Gly327Arg | Missense |
| *HSPB1* | NM_001540.3 | c.16G>A; p.Val6Ile | Missense |
| *HSPB1* | NM_001540.3 | c.24C>T | Synonymous |
| *HSPB1* | NM_001540.3 | c.36G>T | Synonymous |
| *HSPB1* | NM_001540.3 | c.80G>C; p.Arg27Pro | Missense |
| *HSPB1* | NM_001540.3 | c.116C>T; p.Pro39Leu | Missense |
| *HSPB1* | NM_001540.3 | c.142G>A; p.Gly48Ser | Missense |
| *HSPB1* | NM_001540.3 | c.178C>T; p.Pro60Ser | Missense |
| *HSPB1* | NM_001540.3 | c.240A>G | Synonymous |
| *HSPB1* | NM_001540.3 | c.248_249delinsAT; p.Ser83Asn | Missense |
| *HSPB1* | NM_001540.3 | c.277G>A; p.Asp93Asn | Missense |
| *HSPB1* | NM_001540.3 | c.305A>G; p.Asn102Ser | Missense |
| *HSPB1* | NM_001540.3 | c.318G>C | Synonymous |
| *HSPB1* | NM_001540.3 | c.364+6C>G | Intronic |
| *HSPB1* | NM_001540.3 | c.365-14_365-7del | Intronic |
| *HSPB1* | NM_001540.3 | c.365-3A>G | Intronic |
| *HSPB1* | NM_001540.3 | c.365-4A>G | Intronic |
| *HSPB1* | NM_001540.3 | c.365-6C>G | Intronic |
| *HSPB1* | NM_001540.3 | c.365-7C>G | Intronic |
| *HSPB1* | NM_001540.3 | c.376_378del; p.Glu126del | mRNA reading frame maintained |
| *HSPB1* | NM_001540.3 | c.399C>T | Synonymous |
| *HSPB1* | NM_001540.3 | c.404C>G; p.Ser135Cys | Missense |
| *HSPB1* | NM_001540.3 | c.407G>A; p.Arg136Gln | Missense |
| *HSPB1* | NM_001540.3 | c.407G>T; p.Arg136Leu | Missense |
| *HSPB1* | NM_001540.3 | c.415A>G; p.Thr139Ala | Missense |
| *HSPB1* | NM_001540.3 | c.416C>T; p.Thr139Met | Missense |
| *HSPB1* | NM_001540.3 | c.429-7C>T | Intronic |
| *HSPB1* | NM_001540.3 | c.451A>C; p.Thr151Pro | Missense |
| *HSPB1* | NM_001540.3 | c.501C>T | Synonymous |
| *HSPB1* | NM_001540.3 | c.560C>T; p.Ser187Leu | Missense |
| *HSPB1* | NM_001540.3 | c.570G>C; p.Gln190His | Missense |
| *HSPB1* | NM_001540.3 | c.610G>A; p.Ala204Thr | Missense |
| *LITAF* | NM_004862.3 | c.6G>A | Synonymous |
| *LITAF* | NM_004862.3 | c.157G>A; p.Gly53Arg | Missense |
| *LITAF* | NM_004862.3 | c.179C>T; p.Ser60Leu | Missense |
| *LITAF* | NM_004862.3 | c.226G>A; p.Val76Met | Missense |
| *LITAF* | NM_004862.3 | c.232A>G; p.Thr78Ala | Missense |
| *LITAF* | NM_004862.3 | c.240C>T | Synonymous |
| *LITAF* | NM_004862.3 | c.241G>A; p.Val81Met | Missense |
| *LITAF* | NM_004862.3 | c.243G>A | Synonymous |
| *LITAF* | NM_004862.3 | c.327_329dup; p.Asn110dup | mRNA reading frame maintained |
| *LITAF* | NM_004862.3 | c.352T>C; p.Ser118Pro | Missense |
| *LITAF* | NM_004862.3 | c.385G>A; p.Ala129Thr | Missense |
| *LITAF* | NM_004862.3 | c.478C>T; p.Arg160Cys | Missense |
| *LITAF* | NM_004862.3 | c.479G>A; p.Arg160His | Missense |
| *LITAF* | NM_004862.3 | c.*144G>A | 3'UTR |
| *LITAF* | NM_004862.3 | c.*148C>T | 3'UTR |
| *LITAF* | NM_004862.3 | c.*32C>T | 3'UTR |
| *MFN2* | NM_014874.3 | c.20G>A; p.Arg7Gln | Missense |
| *MFN2* | NM_014874.3 | c.33C>T | Synonymous |
| *MFN2* | NM_014874.3 | c.58C>T; p.His20Tyr | Missense |
| *MFN2* | NM_014874.3 | c.72G>T | Synonymous |
| *MFN2* | NM_014874.3 | c.145T>G; p.Tyr49Asp | Missense |
| *MFN2* | NM_014874.3 | c.160G>A; p.Ala54Thr | Missense |
| *MFN2* | NM_014874.3 | c.179C>T; p.Thr60Met | Missense |
| *MFN2* | NM_014874.3 | c.280C>G; p.Arg94Gly | Missense |
| *MFN2* | NM_014874.3 | c.283A>G; p.Arg95Gly | Missense |
| *MFN2* | NM_014874.3 | c.298G>T; p.Ala100Ser | Missense |
| *MFN2* | NM_014874.3 | c.311G>T; p.Arg104Leu | Missense |
| *MFN2* | NM_014874.3 | c.358A>G; p.Lys120Glu | Missense |
| *MFN2* | NM_014874.3 | c.385A>G; p.Thr129Ala | Missense |
| *MFN2* | NM_014874.3 | c.392A>T; p.Asn131Ile | Missense |
| *MFN2* | NM_014874.3 | c.395G>T; p.Cys132Phe | Missense |
| *MFN2* | NM_014874.3 | c.444C>T | Synonymous |
| *MFN2* | NM_014874.3 | c.449G>T; p.Gly150Val | Missense |
| *MFN2* | NM_014874.3 | c.526G>A; p.Gly176Ser | Missense |
| *MFN2* | NM_014874.3 | c.653T>C; p.Leu218Pro | Missense |
| *MFN2* | NM_014874.3 | c.718T>C; p.Phe240Leu | Missense |
| *MFN2* | NM_014874.3 | c.739C>T; p.Arg247Cys | Missense |
| *MFN2* | NM_014874.3 | c.747C>T | Synonymous |
| *MFN2* | NM_014874.3 | c.748C>T; p.Arg250Trp | Missense |
| *MFN2* | NM_014874.3 | c.751C>T; p.Pro251Ser | Missense |
| *MFN2* | NM_014874.3 | c.766C>A; p.Leu256Met | Missense |
| *MFN2* | NM_014874.3 | c.775C>T; p.Arg259Cys | Missense |
| *MFN2* | NM_014874.3 | c.823C>T; p.Arg275Trp | Missense |
| *MFN2* | NM_014874.3 | c.828_830dup; p.Gln276dup | mRNA reading frame maintained |
| *MFN2* | NM_014874.3 | c.838C>T; p.Arg280Cys | Missense |
| *MFN2* | NM_014874.3 | c.933C>T | Synonymous |
| *MFN2* | NM_014874.3 | c.1069A>G; p.Lys357Glu | Missense |
| *MFN2* | NM_014874.3 | c.1078C>G; p.Gln360Glu | Missense |
| *MFN2* | NM_014874.3 | c.1082A>C; p.His361Pro | Missense |
| *MFN2* | NM_014874.3 | c.1085C>G; p.Thr362Arg | Missense |
| *MFN2* | NM_014874.3 | c.1091G>A; p.Arg364Gln | Missense |
| *MFN2* | NM_014874.3 | c.1137_1139delinsCGGGCGGCTCATCAT; p.His380delinsGlyArgLeuIleIle | mRNA reading frame maintained |
| *MFN2* | NM_014874.3 | c.1199G>A; p.Arg400Gln | Missense |
| *MFN2* | NM_014874.3 | c.1202T>G; p.Leu401Arg | Missense |
| *MFN2* | NM_014874.3 | c.1208T>G; p.Phe403Cys | Missense |
| *MFN2* | NM_014874.3 | c.1284G>T; p.Arg428Ser | Missense |
| *MFN2* | NM_014874.3 | c.1413G>A | Synonymous |
| *MFN2* | NM_014874.3 | c.1523C>T; p.Ser508Phe | Missense |
| *MFN2* | NM_014874.3 | c.1528C>T; p.Arg510Trp | Missense |
| *MFN2* | NM_014874.3 | c.1574A>G; p.Asn525Ser | Missense |
| *MFN2* | NM_014874.3 | c.1690C>T; p.Arg564Trp | Missense |
| *MFN2* | NM_014874.3 | c.1759C>A; p.Pro587Thr | Missense |
| *MFN2* | NM_014874.3 | c.1779G>A | Synonymous |
| *MFN2* | NM_014874.3 | c.1879A>G; p.Lys627Glu | Missense |
| *MFN2* | NM_014874.3 | c.1909T>A; p.Ser637Thr | Missense |
| *MFN2* | NM_014874.3 | c.1920C>G | Synonymous |
| *MFN2* | NM_014874.3 | c.1930_1932del; p.Leu644del | mRNA reading frame maintained |
| *MFN2* | NM_014874.3 | c.1946G>A; p.Arg649His | Missense |
| *MFN2* | NM_014874.3 | c.1979C>A; p.Ala660Asp | Missense |
| *MFN2* | NM_014874.3 | c.1981T>C; p.Phe661Leu | Missense |
| *MFN2* | NM_014874.3 | c.1987C>T; p.Arg663Cys | Missense |
| *MFN2* | NM_014874.3 | c.1994T>C; p.Phe665Ser | Missense |
| *MFN2* | NM_014874.3 | c.2119C>T; p.Arg707Trp | Missense |
| *MFN2* | NM_014874.3 | c.2157G>A | Synonymous |
| *MFN2* | NM_014874.3 | c.2170C>G; p.Leu724Val | Missense |
| *MFN2* | NM_014874.3 | c.2200C>G; p.Leu734Val | Missense |
| *MFN2* | NM_014874.3 | c.2222T>G; p.Leu741Trp | Missense |
| *MFN2* | NM_014874.3 | c.2229T>G; p.Ser743Arg | Missense |
| *MFN2* | NM_014874.3 | c.2230G>A; p.Glu744Lys | Missense |
| *MPZ* | NM_000530.6 | c.42G>A | Synonymous |
| *MPZ* | NM_000530.6 | c.77C>T; p.Pro26Leu | Missense |
| *MPZ* | NM_000530.6 | c.88A>G; p.Ile30Val | Missense |
| *MPZ* | NM_000530.6 | c.127G>T; p.Gly43Cys | Missense |
| *MPZ* | NM_000530.6 | c.133C>T; p.Arg45Trp | Missense |
| *MPZ* | NM_000530.6 | c.200G>A; p.Arg67His | Missense |
| *MPZ* | NM_000530.6 | c.216G>A | Synonymous |
| *MPZ* | NM_000530.6 | c.227C>G; p.Ala76Gly | Missense |
| *MPZ* | NM_000530.6 | c.233C>G; p.Ser78Trp | Missense |
| *MPZ* | NM_000530.6 | c.259C>T; p.Pro87Ser | Missense |
| *MPZ* | NM_000530.6 | c.314C>T; p.Pro105Leu | Missense |
| *MPZ* | NM_000530.6 | c.347A>G; p.Asn116Ser | Missense |
| *MPZ* | NM_000530.6 | c.350T>A; p.Leu117Gln | Missense |
| *MPZ* | NM_000530.6 | c.369C>T | Synonymous |
| *MPZ* | NM_000530.6 | c.385G>A; p.Val129Ile | Missense |
| *MPZ* | NM_000530.6 | c.388A>G; p.Lys130Glu | Missense |
| *MPZ* | NM_000530.6 | c.407T>G; p.Val136Gly | Missense |
| *MPZ* | NM_000530.6 | c.409G>T; p.Gly137Cys | Missense |
| *MPZ* | NM_000530.6 | c.448G>C; p.Val150Leu | Missense |
| *MPZ* | NM_000530.6 | c.448G>T; p.Val150Leu | Missense |
| *MPZ* | NM_000530.6 | c.474G>C | Synonymous |
| *MPZ* | NM_000530.6 | c.483G>C | Synonymous |
| *MPZ* | NM_000530.6 | c.509T>G; p.Leu170Arg | Missense |
| *MPZ* | NM_000530.6 | c.527T>C; p.Phe176Ser | Missense |
| *MPZ* | NM_000530.6 | c.585-7_585-5del | Intronic |
| *MPZ* | NM_000530.6 | c.637G>C; p.Gly213Arg | Missense |
| *MPZ* | NM_000530.6 | c.708G>A | Synonymous |
| *NEFL* | NM_006158.3 | c.22C>A; p.Pro8Thr | Missense |
| *NEFL* | NM_006158.3 | c.33G>A | Synonymous |
| *NEFL* | NM_006158.3 | c.65C>T; p.Pro22Leu | Missense |
| *NEFL* | NM_006158.3 | c.217C>G; p.Leu73Val | Missense |
| *NEFL* | NM_006158.3 | c.338_339delinsCC; p.Gln113Pro | Missense |
| *NEFL* | NM_006158.3 | c.418G>A; p.Glu140Lys | Missense |
| *NEFL* | NM_006158.3 | c.471G>A | Synonymous |
| *NEFL* | NM_006158.3 | c.486C>A | Synonymous |
| *NEFL* | NM_006158.3 | c.509C>T; p.Thr170Ile | Missense |
| *NEFL* | NM_006158.3 | c.552C>A; p.Ser184Arg | Missense |
| *NEFL* | NM_006158.3 | c.582A>C; p.Glu194Asp | Missense |
| *NEFL* | NM_006158.3 | c.756C>T | Synonymous |
| *NEFL* | NM_006158.3 | c.793T>G; p.Tyr265Asp | Missense |
| *NEFL* | NM_006158.3 | c.856G>T; p.Val286Leu | Missense |
| *NEFL* | NM_006158.3 | c.893T>C; p.Val298Ala | Missense |
| *NEFL* | NM_006158.3 | c.935T>C; p.Leu312Pro | Missense |
| *NEFL* | NM_006158.3 | c.946A>C; p.Thr316Pro | Missense |
| *NEFL* | NM_006158.3 | c.1026C>G | Synonymous |
| *NEFL* | NM_006158.3 | c.1153G>C; p.Glu385Gln | Missense |
| *NEFL* | NM_006158.3 | c.1157T>G; p.Ile386Ser | Missense |
| *NEFL* | NM_006158.3 | c.1175T>A; p.Leu392His | Missense |
| *NEFL* | NM_006158.3 | c.1185C>T | Synonymous |
| *NEFL* | NM_006158.3 | c.1276G>A; p.Gly426Ser | Missense |
| *NEFL* | NM_006158.3 | c.1319C>T; p.Pro440Leu | Missense |
| *NEFL* | NM_006158.3 | c.1343A>G; p.Gln448Arg | Missense |
| *NEFL* | NM_006158.3 | c.1408C>T; p.Pro470Ser | Missense |
| *NEFL* | NM_006158.3 | c.1461G>A | Synonymous |
| *NEFL* | NM_006158.3 | c.1610A>G; p.Gln537Arg | Missense |
| *PMP22* | NM_000304.2 | c.-6G>T | 5'UTR |
| *PMP22* | NM_000304.2 | c.36C>T | Synonymous |
| *PMP22* | NM_000304.2 | c.49_54dup; p.Val17_Leu18dup | mRNA reading frame maintained |
| *PMP22* | NM_000304.2 | c.55_57del; p.Leu19del | mRNA reading frame maintained |
| *PMP22* | NM_000304.2 | c.68C>A; p.Thr23Lys | Missense |
| *PMP22* | NM_000304.2 | c.82T>C; p.Trp28Arg | Missense |
| *PMP22* | NM_000304.2 | c.103G>A; p.Ala35Thr | Missense |
| *PMP22* | NM_000304.2 | c.125G>A; p.Cys42Tyr | Missense |
| *PMP22* | NM_000304.2 | c.173C>A; p.Pro58Gln | Missense |
| *PMP22* | NM_000304.2 | c.178+5G>A | Intronic |
| *PMP22* | NM_000304.2 | c.292A>G; p.Ile98Val | Missense |
| *PMP22* | NM_000304.2 | c.308A>G; p.Gln103Arg | Missense |
| *PMP22* | NM_000304.2 | c.320-4C>T | Intronic |
| *PMP22* | NM_000304.2 | c.353C>T; p.Thr118Met | Missense |
| *PMP22* | NM_000304.2 | c.418T>C; p.Trp140Arg | Missense |
| *PMP22* | NM_000304.2 | c.440T>C; p.Leu147Pro | Missense |
| *PRX* | NM_181882.2 | c.27+8C>A | Intronic |
| *PRX* | NM_181882.2 | c.80C>A; p.Thr27Asn | Missense |
| *PRX* | NM_181882.2 | c.102A>C | Synonymous |
| *PRX* | NM_181882.2 | c.133C>G; p.Arg45Gly | Missense |
| *PRX* | NM_181882.2 | c.133C>T; p.Arg45Trp | Missense |
| *PRX* | NM_181882.2 | c.331A>G; p.Thr111Ala | Missense |
| *PRX* | NM_181882.2 | c.3324G>A | Synonymous |
| *PRX* | NM_181882.2 | c.361C>T; p.Arg121Trp | Missense |
| *PRX* | NM_181882.2 | c.381+24_381+39dup | Intronic |
| *PRX* | NM_181882.2 | c.445G>A; p.Ala149Thr | Missense |
| *PRX* | NM_181882.2 | c.493C>T; p.Arg165Cys | Missense |
| *PRX* | NM_181882.2 | c.499C>T; p.Arg167Cys | Missense |
| *PRX* | NM_181882.2 | c.500G>C; p.Arg167Pro | Missense |
| *PRX* | NM_181882.2 | c.502C>T; p.Arg168Trp | Missense |
| *PRX* | NM_181882.2 | c.503G>A; p.Arg168Gln | Missense |
| *PRX* | NM_181882.2 | c.554G>A; p.Arg185His | Missense |
| *PRX* | NM_181882.2 | c.580C>T; p.Arg194Cys | Missense |
| *PRX* | NM_181882.2 | c.587G>C; p.Arg196Pro | Missense |
| *PRX* | NM_181882.2 | c.597C>T | Synonymous |
| *PRX* | NM_181882.2 | c.624C>T | Synonymous |
| *PRX* | NM_181882.2 | c.631G>A; p.Ala211Thr | Missense |
| *PRX* | NM_181882.2 | c.716C>T; p.Pro239Leu | Missense |
| *PRX* | NM_181882.2 | c.774C>A | Synonymous |
| *PRX* | NM_181882.2 | c.786A>C | Synonymous |
| *PRX* | NM_181882.2 | c.794G>T; p.Gly265Val | Missense |
| *PRX* | NM_181882.2 | c.840G>A | Synonymous |
| *PRX* | NM_181882.2 | c.847G>A; p.Val283Met | Missense |
| *PRX* | NM_181882.2 | c.901C>T; p.Pro301Ser | Missense |
| *PRX* | NM_181882.2 | c.941C>T; p.Thr314Ile | Missense |
| *PRX* | NM_181882.2 | c.1026A>C | Synonymous |
| *PRX* | NM_181882.2 | c.1091G>A; p.Arg364Gln | Missense |
| *PRX* | NM_181882.2 | c.1129G>T; p.Val377Leu | Missense |
| *PRX* | NM_181882.2 | c.1251G>T | Synonymous |
| *PRX* | NM_181882.2 | c.1397C>T; p.Pro466Leu | Missense |
| *PRX* | NM_181882.2 | c.1460C>T; p.Pro487Leu | Missense |
| *PRX* | NM_181882.2 | c.1490_1567del; p.Pro497_Leu522del | mRNA reading frame maintained |
| *PRX* | NM_181882.2 | c.1509A>T; p.Lys503Pro | Missense |
| *PRX* | NM_181882.2 | c.1539G>A | Synonymous |
| *PRX* | NM_181882.2 | c.1547G>A; p.Arg516Gln | Missense |
| *PRX* | NM_181882.2 | c.1568_1645del; p.Leu523_Leu548del | mRNA reading frame maintained |
| *PRX* | NM_181882.2 | c.1578G>A | Synonymous |
| *PRX* | NM_181882.2 | c.1714G>A; p.Val572Met | Missense |
| *PRX* | NM_181882.2 | c.1749T>G | Synonymous |
| *PRX* | NM_181882.2 | c.1752G>C; p.Glu584Asp | Missense |
| *PRX* | NM_181882.2 | c.1775A>C; p.Glu592Ala | Missense |
| *PRX* | NM_181882.2 | c.1828G>A; p.Glu610Lys | Missense |
| *PRX* | NM_181882.2 | c.1837G>A; p.Val613Met | Missense |
| *PRX* | NM_181882.2 | c.1845T>C | Synonymous |
| *PRX* | NM_181882.2 | c.1951G>A; p.Asp651Asn | Missense |
| *PRX* | NM_181882.2 | c.1957C>T; p.His653Tyr | Missense |
| *PRX* | NM_181882.2 | c.1964C>T; p.Pro655Leu | Missense |
| *PRX* | NM_181882.2 | c.1979C>G; p.Pro660Arg | Missense |
| *PRX* | NM_181882.2 | c.2044G>A; p.Gln682Lys | Missense |
| *PRX* | NM_181882.2 | c.2080C>T; p.Pro694Ser | Missense |
| *PRX* | NM_181882.2 | c.2215G>C; p.Asp739His | Missense |
| *PRX* | NM_181882.2 | c.2229C>T | Synonymous |
| *PRX* | NM_181882.2 | c.2254G>A; p.Glu752Lys | Missense |
| *PRX* | NM_181882.2 | c.2274G>T; p.Met758Ile | Missense |
| *PRX* | NM_181882.2 | c.2285A>G; p.Lys762Arg | Missense |
| *PRX* | NM_181882.2 | c.2295C>T | Synonymous |
| *PRX* | NM_181882.2 | c.2335G>A; p.Ala779Thr | Missense |
| *PRX* | NM_181882.2 | c.2383A>G; p.Met795Val | Missense |
| *PRX* | NM_181882.2 | c.2449C>T; p.Arg817Cys | Missense |
| *PRX* | NM_181882.2 | c.2464G>A; p.Glu822Lys | Missense |
| *PRX* | NM_181882.2 | c.2520G>C; p.Glu840Asp | Missense |
| *PRX* | NM_181882.2 | c.2548C>G; p.Pro850Ala | Missense |
| *PRX* | NM_181882.2 | c.2641C>T; p.Arg881Trp | Missense |
| *PRX* | NM_181882.2 | c.2677G>A; p.Glu893Lys | Missense |
| *PRX* | NM_181882.2 | c.2728G>A; p.Ala910Thr | Missense |
| *PRX* | NM_181882.2 | c.2731G>A; p.Val911Met | Missense |
| *PRX* | NM_181882.2 | c.2817G>A | Synonymous |
| *PRX* | NM_181882.2 | c.2832T>C | Synonymous |
| *PRX* | NM_181882.2 | c.2837C>T; p.Ala946Val | Missense |
| *PRX* | NM_181882.2 | c.2907T>C | Synonymous |
| *PRX* | NM_181882.2 | c.2968G>A; p.Asp990Asn | Missense |
| *PRX* | NM_181882.2 | c.2999C>T; p.Ala1000Val | Missense |
| *PRX* | NM_181882.2 | c.3037G>A; p.Asp1013Asn | Missense |
| *PRX* | NM_181882.2 | c.3110A>G; p.Glu1037Gly | Missense |
| *PRX* | NM_181882.2 | c.3209G>A; p.Arg1070Gln | Missense |
| *PRX* | NM_181882.2 | c.3291C>A | Synonymous |
| *PRX* | NM_181882.2 | c.3496C>T; p.Pro1166Ser | Missense |
| *PRX* | NM_181882.2 | c.3549C>T | Synonymous |
| *PRX* | NM_181882.2 | c.3656C>T; p.Pro1219Leu | Missense |
| *PRX* | NM_181882.2 | c.3673G>A; p.Val1225Met | Missense |
| *PRX* | NM_181882.2 | c.3708G>A | Synonymous |
| *PRX* | NM_181882.2 | c.3769G>A; p.Gly1257Arg | Missense |
| *PRX* | NM_181882.2 | c.3772G>T; p.Gly1258Cys | Missense |
| *PRX* | NM_181882.2 | c.3838G>C; p.Glu1280Gln | Missense |
| *PRX* | NM_181882.2 | c.3886G>C; p.Gly1296Arg | Missense |
| *PRX* | NM_181882.2 | c.3935G>T; p.Gly1312Val | Missense |
| *PRX* | NM_181882.2 | c.3947C>T; p.Ala1316Val | Missense |
| *PRX* | NM_181882.2 | c.3960C>T | Synonymous |
| *PRX* | NM_181882.2 | c.4004G>A; p.Arg1335Gln | Missense |
| *PRX* | NM_181882.2 | c.4074_4079del; p.Glu1360_Glu1361del | mRNA reading frame maintained |
| *PRX* | NM_181882.2 | c.4108C>A; p.Arg1370Ser | Missense |
| *PRX* | NM_181882.2 | c.4112G>A; p.Arg1371Gln | Missense |
| *PRX* | NM_181882.2 | c.4118G>A; p.Arg1373Gln | Missense |
| *PRX* | NM_181882.2 | c.4121T>G; p.Val1374Gly | Missense |
| *PRX* | NM_181882.2 | c.4129C>T; p.Arg1377Cys | Missense |
| *PRX* | NM_181882.2 | c.4152G>A | Synonymous |
| *PRX* | NM_181882.2 | c.4157C>A; p.Pro1386His | Missense |
| *PRX* | NM_181882.2 | c.4165G>C; p.Ala1389Pro | Missense |
| *PRX* | NM_181882.2 | c.4171C>G; p.Arg1391Gly | Missense |
| *PRX* | NM_181882.2 | c.4171C>T; p.Arg1391Trp | Missense |
| *PRX* | NM_181882.2 | c.4174G>A; p.Gly1392Arg | Missense |
| *PRX* | NM_181882.2 | c.4219T>G; p.Ser1407Ala | Missense |
| *PRX* | NM_181882.2 | c.4256C>T; p.Pro1419Leu | Missense |
| *PRX* | NM_181882.2 | c.4300C>T; p.Arg1434Trp | Missense |
| *PRX* | NM_181882.2 | c.4307G>A; p.Arg1436Gln | Missense |
| *RAB7A* | NM_004637.5 | c.87G>A | Synonymous |
| *RAB7A* | NM_004637.5 | c.89A>G; p.Asn30Ser | Missense |
| *RAB7A* | NM_004637.5 | c.213G>A | Synonymous |
| *RAB7A* | NM_004637.5 | c.377A>G; p.Lys126Arg | Missense |
| *SH3TC2* | NM_024577.3 | c.32G>A; p.Arg11Gln | Missense |
| *SH3TC2* | NM_024577.3 | c.46G>A; p.Gly16Ser | Missense |
| *SH3TC2* | NM_024577.3 | c.82G>C; p.Val28Leu | Missense |
| *SH3TC2* | NM_024577.3 | c.188G>A; p.Arg63Lys | Missense |
| *SH3TC2* | NM_024577.3 | c.238G>A; p.Ala80Thr | Missense |
| *SH3TC2* | NM_024577.3 | c.265C>T; p.Arg89Cys | Missense |
| *SH3TC2* | NM_024577.3 | c.280-4G>A | Intronic |
| *SH3TC2* | NM_024577.3 | c.385+4_385+8del | Intronic |
| *SH3TC2* | NM_024577.3 | c.469C>A; p.Gln157Lys | Missense |
| *SH3TC2* | NM_024577.3 | c.505T>C; p.Tyr169His | Missense |
| *SH3TC2* | NM_024577.3 | c.549C>T | Synonymous |
| *SH3TC2* | NM_024577.3 | c.581A>G; p.Glu194Gly | Missense |
| *SH3TC2* | NM_024577.3 | c.635A>G; p.Glu212Gly | Missense |
| *SH3TC2* | NM_024577.3 | c.679C>T; p.Arg227Trp | Missense |
| *SH3TC2* | NM_024577.3 | c.689T>C; p.Val230Ala | Missense |
| *SH3TC2* | NM_024577.3 | c.706G>A; p.Glu236Lys | Missense |
| *SH3TC2* | NM_024577.3 | c.741A>G | Synonymous |
| *SH3TC2* | NM_024577.3 | c.751C>T; p.Pro251Ser | Missense |
| *SH3TC2* | NM_024577.3 | c.766C>T; p.Leu256Phe | Missense |
| *SH3TC2* | NM_024577.3 | c.830C>T; p.Thr277Met | Missense |
| *SH3TC2* | NM_024577.3 | c.908C>A; p.Pro303His | Missense |
| *SH3TC2* | NM_024577.3 | c.929G>A; p.Gly310Glu | Missense |
| *SH3TC2* | NM_024577.3 | c.1002-7G>A | Intronic |
| *SH3TC2* | NM_024577.3 | c.1177+10G>A | Intronic |
| *SH3TC2* | NM_024577.3 | c.1177+9C>T | Intronic |
| *SH3TC2* | NM_024577.3 | c.1178C>T; p.Ala393Val | Missense |
| *SH3TC2* | NM_024577.3 | c.1211C>T; p.Pro404Leu | Missense |
| *SH3TC2* | NM_024577.3 | c.1253A>G; p.Gln418Arg | Missense |
| *SH3TC2* | NM_024577.3 | c.1254G>T; p.Gln418His | Missense |
| *SH3TC2* | NM_024577.3 | c.1298C>T; p.Ser433Leu | Missense |
| *SH3TC2* | NM_024577.3 | c.1342G>C; p.Asp448His | Missense |
| *SH3TC2* | NM_024577.3 | c.137A>C; p.Gln46Pro | Missense |
| *SH3TC2* | NM_024577.3 | c.1469_1471del; p.Ser490del | mRNA reading frame maintained |
| *SH3TC2* | NM_024577.3 | c.1483A>G; p.Thr495Ala | Missense |
| *SH3TC2* | NM_024577.3 | c.1513G>A; p.Asp505Asn | Missense |
| *SH3TC2* | NM_024577.3 | c.1522G>A; p.Val508Met | Missense |
| *SH3TC2* | NM_024577.3 | c.1585C>T; p.Arg529Trp | Missense |
| *SH3TC2* | NM_024577.3 | c.1619G>A; p.Arg540Lys | Missense |
| *SH3TC2* | NM_024577.3 | c.1721A>G; p.Asn574Ser | Missense |
| *SH3TC2* | NM_024577.3 | c.1767C>T | Synonymous |
| *SH3TC2* | NM_024577.3 | c.1862G>A; p.Arg621His | Missense |
| *SH3TC2* | NM_024577.3 | c.1921C>T; p.Arg641Cys | Missense |
| *SH3TC2* | NM_024577.3 | c.1942C>T; p.Arg648Trp | Missense |
| *SH3TC2* | NM_024577.3 | c.2097G>A | Synonymous |
| *SH3TC2* | NM_024577.3 | c.2189G>A; p.Gly730Asp | Missense |
| *SH3TC2* | NM_024577.3 | c.2218C>T; p.Leu740Phe | Missense |
| *SH3TC2* | NM_024577.3 | c.2254G>A; p.Asp752Asn | Missense |
| *SH3TC2* | NM_024577.3 | c.2304C>T | Synonymous |
| *SH3TC2* | NM_024577.3 | c.2339T>C; p.Leu780Pro | Missense |
| *SH3TC2* | NM_024577.3 | c.2352G>T; p.Leu784Phe | Missense |
| *SH3TC2* | NM_024577.3 | c.2520C>T | Synonymous |
| *SH3TC2* | NM_024577.3 | c.2528G>T; p.Gly843Val | Missense |
| *SH3TC2* | NM_024577.3 | c.2552G>A; p.Arg851Gln | Missense |
| *SH3TC2* | NM_024577.3 | c.2785G>C; p.Val929Leu | Missense |
| *SH3TC2* | NM_024577.3 | c.2858A>C; p.His953Pro | Missense |
| *SH3TC2* | NM_024577.3 | c.2873-4T>A | Intronic |
| *SH3TC2* | NM_024577.3 | c.2950C>A; p.His984Asn | Missense |
| *SH3TC2* | NM_024577.3 | c.2954A>G; p.Glu985Gly | Missense |
| *SH3TC2* | NM_024577.3 | c.3049G>T; p.Ala1017Ser | Missense |
| *SH3TC2* | NM_024577.3 | c.3094C>T; p.Arg1032Cys | Missense |
| *SH3TC2* | NM_024577.3 | c.3127G>T; p.Ala1043Ser | Missense |
| *SH3TC2* | NM_024577.3 | c.3166C>T; p.Leu1056Phe | Missense |
| *SH3TC2* | NM_024577.3 | c.3183G>A | Synonymous |
| *SH3TC2* | NM_024577.3 | c.3294C>T | Synonymous |
| *SH3TC2* | NM_024577.3 | c.3320A>G; p.Tyr1107Cys | Missense |
| *SH3TC2* | NM_024577.3 | c.3332G>T; p.Gly1111Val | Missense |
| *SH3TC2* | NM_024577.3 | c.3380G>A; p.Arg1127Gln | Missense |
| *SH3TC2* | NM_024577.3 | c.3418G>A; p.Glu1140Lys | Missense |
| *SH3TC2* | NM_024577.3 | c.3433G>T; p.Ala1145Ser | Missense |
| *SH3TC2* | NM_024577.3 | c.3479-8A>G | Intronic |
| *SH3TC2* | NM_024577.3 | c.3511C>T; p.Arg1171Cys | Missense |
| *SH3TC2* | NM_024577.3 | c.3522A>G | Synonymous |
| *SH3TC2* | NM_024577.3 | c.3550A>G; p.Met1184Val | Missense |
| *SH3TC2* | NM_024577.3 | c.3722C>T; p.Ala1241Val | Missense |
| *SH3TC2* | NM_024577.3 | c.3813C>G | Synonymous |
| *SH3TC2* | NM_024577.3 | c.3835C>T; p.Arg1279Trp | Missense |

Abbreviations: *PMP22 =* peripheral myelin protein 22; *GJB1 =* gap junction protein, beta-1; *MPZ =* myelin protein zero; *MFN2 =* mitofusin 2*; SH3TC2 =* SH3 domain and tetratricopeptide repeat domain 2; *GDAP1 =* ganglioside-induced differentiation-associated protein; *NEFL =* neurofilament protein light polypeptide; *LITAF =* lipopolysaccharide-induced tumor necrosis factor-alpha factor; *GARS =* glycyl-tRNA synthetase, *HSPB1* heat-shock 27-kd protein 1; *FIG4 =* S. Cerevisiae homolog of fig4; *EGR2* = early growth response 2; *PRX =* periaxin*; RAB7A =* RAS-associated protein RAB7.

**Supplemental Table 3.** The Positive Rate for Charcot-Marie-Tooth Disease Gene Mutations and Non-synonymous Variants as Determined by MLPA and Sanger DNA sequencing (n= 17,377).

| Gene | Positive Rate% (n) | Non-Synonymous VUS % (n) |
| --- | --- | --- |
| *PMP22 dup* | 10.49 (1,822) | NA |
| *PMP22 del* | 4.06 (705) | NA |
| *GJB1* | 1.23 (214) | 0.37 (64) |
| *MPZ* | 0.97 (169) | 0.55 (96) |
| *MFN2* | 0.79 (137) | 1.12 (194) |
| *PMP22* | 0.16 (28) | 0.77 (134) |
| *SH3TC2* | 0.16 (27) | 1.97 (343) |
| *GDAP1* | 0.14 (24) | 0.45 (78) |
| *NEFL* | 0.13 (23) | 0.55 (96) |
| *LITAF* | 0.10 (17) | 0.28 (49) |
| *GARS* | 0.07 (12) | 0.42 (73) |
| *HSPB1* | 0.06 (11) | 0.56 (97) |
| *FIG4* | 0.06 (10) | 0.27 (47) |
| *GJB1 del* | 0.06 (10) | NA |
| *EGR2* | 0.02 (4) | 0.23 (40) |
| *PRX* | 0.01 (1) | 2.62 (456) |
| *RAB7A* | 0.01 (2) | 0.03 (6) |

Abbreviations: MLPA = multiplex ligation-dependent probe amplification; VUS = variant of unknown significance; dup = duplication; del = deletion; NA = not available; *PMP22 =* peripheral myelin protein 22; *GJB1 =* gap junction protein, beta-1; *MPZ =* myelin protein zero; *MFN2 =* mitofusin 2*; SH3TC2 =* SH3 domain and tetratricopeptide repeat domain 2; *GDAP1 =* ganglioside-induced differentiation-associated protein; *NEFL =* neurofilament protein light polypeptide; *LITAF =* lipopolysaccharide-induced tumor necrosis factor-alpha factor; *GARS =* glycyl-tRNA synthetase, *HSPB1* heat-shock 27-kd protein 1; *FIG4 =* S. Cerevisiae homolog of fig4; *EGR2* = early growth response 2; *PRX =* periaxin*; RAB7A =* RAS-associated protein RAB7.

**Supplemental Table 4.** The Positive Rate for Charcot-Marie-Tooth Disease Gene Mutations and Non-synonymous Variants as Determined by MLPA and NGS (n= 503).

| Gene | Positive Rate % (n) | Non-Synonymous VUS % (n) |
| --- | --- | --- |
| *PMP22 dup* | 12.33 (62) | NA |
| *PMP22 del* | 3.18 (16) | NA |
| *MFN2* | 1.19 (6) | 2.19 (11) |
| *GJB1* | 0.99 (5) | 0.20 (1) |
| *PMP22* | 0.40 (2) | 0.40 (2) |
| *MPZ* | 0.40 (2) | 0.20 (1) |
| *LITAF* | 0.20 (1) | 0.00 (0) |
| *GDAP1* | 0.20 (1) | 0.40 (2) |
| *SH3TC2* | 0.00 (0) | 0.40 (2) |
| *GARS* | 0.00 (0) | 0.40 (2) |
| *NEFL* | 0.00 (0) | 0.00 (0) |
| *HSPB1* | 0.00 (0) | 0.99 (5) |
| *FIG4* | 0.00 (0) | 0.00 (0) |
| *GDAP1* | 0.20 (1) | 0.40 (2) |
| *PRX* | 0.00 (0) | 1.79 (9) |
| *EGR2* | 0.00 (0) | 0.00 (0) |
| *RAB7A* | 0.00 (0) | 0.20 (1) |
| *GJB1 del* | 0.00 (0) | NA |

Abbreviations: MLPA = multiplex ligation-dependent probe amplification; NGS = next generation sequencing; VUS = variant of unknown significance; dup = duplication; del = deletion; NA = not available; *PMP22 =* peripheral myelin protein 22; *GJB1 =* gap junction protein, beta-1; *MPZ =* myelin protein zero; *MFN2 =* mitofusin 2*; SH3TC2 =* SH3 domain and tetratricopeptide repeat domain 2; *GDAP1 =* ganglioside-induced differentiation-associated protein; *NEFL =* neurofilament protein light polypeptide; *LITAF =* lipopolysaccharide-induced tumor necrosis factor-alpha factor; *GARS =* glycyl-tRNA synthetase, *HSPB1* heat-shock 27-kd protein 1; *FIG4 =* S. Cerevisiae homolog of fig4; *EGR2* = early growth response 2; *PRX =* periaxin*; RAB7A =* RAS-associated protein RAB7.

**Supplemental Table 5.** Previously Unpublished Pathogenic Charcot-Marie-Tooth Mutations Detected in this Study (n=87).

| Gene | Reference Sequence | Variant | Mutation Type |
| --- | --- | --- | --- |
| *MFN2* | NM_014874.3 | c.30dup; p.Ile11Tyrfs*8 | Frameshift |
|  |  | c.1392+1G>A | Splice Site |
|  |  | c.1772_1773del; p.Gln591Argfs*57 | Frameshift |
|  |  | c.2037C>G; p.Y679* | Nonsense |
|  |  | c.312-1G>T | Splice Site |
|  |  | c.669T>G; p.F223L | Missense |
| *GJB1* | NM_000166.5 | c.3G>C; p.M1I | Missense (initiator) |
|  |  | c.498_502del; p.Lys167Argfs*74 | Frameshift |
|  |  | c.576del; p.Phe193Serfs*3 | Frameshift |
|  |  | c.622del; p.Glu208Argfs*45 | Frameshift |
|  |  | c.629_632del; p.Val210Alafs*42 | Frameshift |
|  |  | c.772del; p.Ser258Profs*2 | Frameshift |
|  |  | c.785_786del; p.Ile262Thrfs*13 | Frameshift |
|  |  | c.800del; p.Pro267Leufs*125 | Frameshift |
|  |  | c.822del; p.Glu275Lysfs*117 | Frameshift |
|  |  | c.844dup; p.Ala282Glyfs*129 | Frameshift |
|  |  | c.851G>C; p.*234S | Non-stop |
|  |  | c.852A>G; p.*284W | Non-stop |
| *FIG4* | NM_014845.5 | c.1149_1150delinsT; p.Lys383Asnfs*8 | Frameshift |
|  |  | c.1239_1241delinsATA; p.Y413-414* | Nonsense |
|  |  | c.2299dup; p.Glu767Glyfs*17 | Frameshift |
|  |  | c.2386C>T; p.Q796* | Nonsense |
|  |  | c.2459+1G>A | Splice Site |
|  |  | c.2547-1G>A | Splice Site |
|  |  | c.737G>A; p.W246* | Nonsense |
| *GARS* | NM_002047.2 | c.1809+1G>A | Splice Site |
|  |  | c.1896_1903+3delins9 | Splice Site |
|  |  | c.1031+1G>A | Splice Site |
| *GDAP1* | NM_018972.2 | c.1A>T; p.M1L | Missense (initiator) |
|  |  | c.116del; p.Lys39Argfs*5 | Frameshift |
|  |  | c.501del; p.Glu168Serfs*4 | Frameshift |
|  |  | c.577A>T; p.K193* | Nonsense |
|  |  | c.579del; p.Lys193Asnfs*13 | Frameshift |
|  |  | c.1019dup; p.Arg341Glnfs*12 | Frameshift |
|  |  | c.714G>A; p.W238* | Nonsense |
|  |  | c.817C>T; p.R273* | Nonsense |
|  |  | c.928del; p.Arg310Glyfs*38 | Frameshift |
| *HSPB1* | NM_001540.3 | c.171_172insGCGCCCT; p.Leu58Alafs*105 | Frameshift |
|  |  | c.505del; p.Met169Cysfs*4 | Frameshift |
| *MPZ* | NM_000530.6 | c.1A>G; p.M1V | Missense (initiator) |
|  |  | c.129_136del; p.Ser44Aspfs*10 | Frameshift |
|  |  | c.197G>A; p.W66* | Nonsense |
|  |  | c.204C>A; p.Y68* | Nonsense |
|  |  | c.68-5_71del | Splice Site |
|  |  | c.256C>T; p.Q86* | Nonsense |
|  |  | c.263_264insACCCTA; p.Tyr88* | Frameshift |
|  |  | c.434_437del; p.Tyr145Serfs*16 | Frameshift |
|  |  | c.235-2A>C | Splice Site |
|  |  | c.448+1G>A | Splice Site |
|  |  | c.448+2T>G | Splice Site |
|  |  | c.486del; p.Ile162Metfs*90 | Frameshift |
|  |  | c.560del; p.Gln187Argfs*65 | Frameshift |
|  |  | c.571C>T; p.Q191* | Nonsense |
|  |  | c.574_575del; p.Arg192Glufs*42 | Frameshift |
|  |  | c.646dup; p.Thr216Asnfs*19 | Frameshift |
|  |  | c.745T>C; p.*249Q | Non-stop |
|  |  | c.646-10_650del | Splice Site |
| *NEFL* | NM_006158.2 | c.556G>T; p.E186* | Nonsense |
|  |  | c.1261C>T; p.R421* | Nonsense |
| *PMP22* | NM_000304.3 | c.76del; p.Ser26Alafs*44 | Frameshift |
|  |  | c.78+1G>A | Splice Site |
|  |  | c.78+1G>C | Splice Site |
|  |  | c.138del; p.Ser47Glnfs*23 | Frameshift |
|  |  | c.138_139delinsA; p.Ser47Glnfs*23 | Frameshift |
|  |  | c.319+1G>T | Splice Site |
|  |  | c.383C>A; p.S128* | Nonsense |
| *PRX* | NM_181882.2 | c.124_125dup; p.Phe43Serfs*25 | Frameshift |
|  |  | c.231C>G; p.Y77* | Nonsense |
|  |  | c.1173del; p.Arg392Glufs*20 | Frameshift |
|  |  | c.1864C>T; p.Q622* | Nonsense |
|  |  | c.2689C>T; p.R897* | Nonsense |
|  |  | c.3685C>T; p.R1229* | Nonsense |
|  |  | c.4003C>T; p.R1335* | Nonsense |
| *SH3TC2* | NM_024577.3 | c.1378C>T; p.Q460* | Nonsense |
|  |  | c.1384G>T; p.E462* | Nonsense |
|  |  | c.2989del; p.Arg997Glyfs*18 | Frameshift |
|  |  | c.3154C>T; p.R1052* | Nonsense |
|  |  | c.3303del; p.Arg1101Serfs*15 | Frameshift |
|  |  | c.3627T>A; p.Y1209* | Nonsense |
|  |  | c.375C>A; p.Y125* | Nonsense |
|  |  | c.386-2A>C | Splice Site |
|  |  | c.524del; p.Gln175Argfs*13 | Frameshift |
|  |  | c.688del; p.Val230Tyrfs*18 | Frameshift |
|  |  | c.735G>A; p.W245* | Nonsense |
|  |  | c.805+2T>C | Splice Site |
|  |  | c.957del; p.Phe320Leufs*7 | Frameshift |
|  |  | c.806-2A>G | Splice Site |

Abbreviations: *PMP22 =* peripheral myelin protein 22; *GJB1 =* gap junction protein, beta-1; *MPZ =* myelin protein zero; *MFN2 =* mitofusin 2*; SH3TC2 =* SH3 domain and tetratricopeptide repeat domain 2; *GDAP1 =* ganglioside-induced differentiation-associated protein; *NEFL =* neurofilament protein light polypeptide; *LITAF =* lipopolysaccharide-induced tumor necrosis factor-alpha factor; *GARS =* glycyl-tRNA synthetase, *HSPB1* heat-shock 27-kd protein 1; *FIG4 =* S. Cerevisiae homolog of fig4; *EGR2* = early growth response 2; *PRX =* periaxin*; RAB7A =* RAS-associated protein RAB7.

**Supplemental Table 6.** The Top Five (20.3%) Recurring Charcot-Marie-Tooth Mutations^a^ in this study.

| Variant | % of all pathogenic mutations |
| --- | --- |
| *MFN2*:c.2219G>C; p.W740S | 5.4% |
| *SH3TC2*:c.2860C>T; p.R954* | 4.8% |
| *FIG4*:c.122T>C; p.I41T | 4.3% |
| *MFN2*:c.227T>C; p.L76P | 3.3% |
| *GJB1*:c.305A>G; p.E102G | 2.5% |

^a^The data for *FIG4* and *SH3TC2* include carrier status patients and positive patients.

Abbreviations: *MFN2 =* mitofusin 2*; SH3TC2 =* SH3 domain and tetratricopeptide repeat domain 2; *FIG4 =* S. Cerevisiae homolog of fig4; *GJB1 =* gap junction protein, beta-1.

**Supplemental Table** 7. A Comparison of Positive Rates Before and After the Introduction of NGS.

| Gene/Test | Sanger Positive Result Frequency (%) | Next Generation Sequencing Positive Result Frequency (%) | P value* |
| --- | --- | --- | --- |
| *PMP22 dup* | 10.49 | 12.33 | 0.1144 |
| *PMP22 del* | 4.06 | 3.18 | 0.2587 |
| *GJB1* | 1.23 | 0.99 | 0.8329 |
| *MPZ* | 0.97 | 0.40 | 0.2374 |
| *MFN2* | 0.79 | 1.19 | 0.3025 |
| *SH3TC2* | 0.16 | 0.00 | 1.0000 |
| *PMP22* | 0.16 | 0.40 | 0.2121 |
| *GDAP1* | 0.14 | 0.20 | 0.5183 |
| *NEFL* | 0.13 | 0.00 | 1.0000 |
| *LITAF* | 0.10 | 0.20 | 0.0926 |
| *GARS* | 0.07 | 0.00 | 1.0000 |
| *HSPB1* | 0.06 | 0.00 | 1.0000 |
| *FIG4* | 0.06 | 0.00 | 1.0000 |
| *GJB1 del* | 0.06 | 0.00 | 1.0000 |
| *EGR2* | 0.02 | 0.00 | 0.1100 |
| *PRX* | 0.01 | 0.00 | 1.0000 |
| *RAB7A* | 0.01 | 0.00 | 1.0000 |

*Fisher’s two-tailed exact test.

Abbreviations: *PMP22 =* peripheral myelin protein 22; *GJB1 =* gap junction protein, beta-1; *MPZ =* myelin protein zero; *MFN2 =* mitofusin 2*; SH3TC2 =* SH3 domain and tetratricopeptide repeat domain 2; *GDAP1 =* ganglioside-induced differentiation-associated protein; *NEFL =* neurofilament protein light polypeptide; *LITAF =* lipopolysaccharide-induced tumor necrosis factor-alpha factor; *GARS =* glycyl-tRNA synthetase, *HSPB1* heat-shock 27-kd protein 1; *FIG4 =* S. Cerevisiae homolog of fig4; *EGR2* = early growth response 2; *PRX =* periaxin*; RAB7A =* RAS-associated protein RAB7.
